# Correction: Quaresima et al. New Insights in the Setting of Transplant Oncology. *Medicina* 2023, *59*, 568

**DOI:** 10.3390/medicina61122242

**Published:** 2025-12-18

**Authors:** Silvia Quaresima, Fabio Melandro, Francesco Giovanardi, Kejal Shah, Valerio De Peppo, Gianluca Mennini, Davide Ghinolfi, Ashley Limkemann, Timothy M. Pawlik, Quirino Lai

**Affiliations:** 1General Surgery and Organ Transplantation Unit, AOU Policlinico Umberto I, Sapienza University of Rome, 00161 Rome, Italy; silvia.quaresima@uniroma1.it (S.Q.); fabio.melandro@uniroma1.it (F.M.); giovanardi89@gmail.com (F.G.); valeriodepeppo2794@gmail.com (V.D.P.); gianluca.mennini@uniroma1.it (G.M.); 2Department of Surgery, Division of Surgical Oncology, James Cancer Center, The Ohio State University, Columbus, OH 43210, USA; kejal.saha@osumc.edu (K.S.); ashley.limkemann@osumc.edu (A.L.); tim.pawlik@osumc.edu (T.M.P.); 3Hepatobiliary Surgery and Liver Transplantation, University of Pisa Medical School Hospital, 56124 Pisa, Italy; d.ghinolfi@ao-pisa.toscana.it

## Text Correction

There was an error in the original publication [1]. Authors cited the wrong reference that introduced the concept of “transplant oncology” for the first time. 

A correction has been made to the Introduction:

In 2014, Dr. Hibi first introduced the concept of “transplant oncology” to describe the intersection of oncologic management and liver transplantation [6]. 

The authors state that the scientific conclusions are unaffected. This correction was approved by the Academic Editor. The original publication has also been updated.

## References

The original reference [6] has been replaced in the corrected manuscript as follows:
6.Hibi, T.; Shinoda, M.; Itano, O.; Kitagawa, Y. Current status of the organ replacement approach for malignancies and an overture for organ bioengineering and regenerative medicine. *Organogenesis* **2014**, *10*, 241–249.

The authors state that the scientific conclusions are unaffected. This correction was approved by the Academic Editor. The original publication has also been updated.
